# Feasibility of a remote, automated daily delivery verification of volumetric‐modulated arc therapy treatments using a commercial record and verify system

**DOI:** 10.1120/jacmp.v13i2.3606

**Published:** 2012-03-08

**Authors:** Jonas D. Fontenot

**Affiliations:** ^1^ Department of Physics Mary Bird Perkins Cancer Center Baton Rouge Louisiana; ^2^ Department of Physics and Astronomy Louisiana State University and Agricultural and Mechanical College Baton Rouge Louisiana USA

**Keywords:** volumetric‐modulated arc therapy, quality assurance, record and verify systems

## Abstract

Volumetric‐modulated arc therapy (VMAT) is an effective but complex technique for delivering radiation therapy. VMAT relies on precise combinations of dose rate, gantry speed, and multileaf collimator (MLC) shapes to deliver intensity‐modulated patterns. Such complexity warrants the development of correspondingly robust performance verification systems. In this work, we report on a remote, automated software system for daily delivery verification of VMAT treatments. The performance verification software system consists of three main components: (1) a query module for retrieving daily MLC, gantry, and jaw positions reported by the linear accelerator control system to the record and verify system; (2) an analysis module which reads the daily delivery report generated from the database query module, compares the reported treatment positions against the planned positions, and compiles delivery position error reports; and (3) a graphical reporting module which displays reports initiated by a user anywhere within the institutional network or which can be configured to alert authorized users when predefined tolerance values are exceeded. The utility of the system was investigated through analysis of patient data collected at our clinic. Nearly 2500 VMAT fractions have been analyzed with the delivery verification system at our institution. The average percentage of reported MLC leaf positions within 3 mm, gantry positions within 2°, and jaw positions within 3 mm of their planned positions was 92.9%±5.5%,95.9%±2.9%, and 99.7%±0.6%, respectively. The level of agreement between planned and reported MLC positions decreased for treatment plans requiring larger MLC leaf movements between control points. Differences in the reported MLC position error between the delivery verification system and data extracted manually from the control system were noted; however, the differences are likely systematic and, therefore, may be characterized if appropriately accounted for. Further investigation is needed to confirm the utility and accuracy of the system.

PACS numbers: 87.55.N‐, 87.55.T‐, 87.55.Qr

## I. INTRODUCTION

Radiation therapy delivery techniques are becoming increasingly complex. As has been underscored by recent media publicity,^(^ ^1^ ^–^ ^3^ ^)^ the need for systems that ensure patient safety during treatment is also growing. Although manufacturers of linear accelerators have integrated safety features into their products, there is strong motivation to introduce independent mechanisms to verify accurate delivery in the event that manufacturer systems fail or malfunction.

Volumetric‐modulated arc therapy (VMAT) represents one of the most recent and complex radiation delivery techniques to date. Numerous recent reports in the literature have indicated the potential of VMAT to improve treatment plan quality and dramatically decrease treatment times relative to fixed‐beam IMRT.^(^ ^4^ ^–^ ^11^ ^)^ VMAT treatments rely on precise, time‐dependent combinations of gantry speed, dose rate, and multileaf collimator (MLC) positions to produce planned intensity patterns.^(7)^ Such requirements necessitate correspondingly precise and reliable performance from the linear accelerator system, with failure to achieve the necessary parameters resulting in potentially dire dosimetric consequences. Thus, the degree of complexity associated with these precise combinations justifies the introduction of correspondingly robust performance verification systems.

Recently, a number of delivery verification solutions have been described. For example, the performance of the linear accelerator during delivery of rotational therapy has been verified using transmission measurements acquired with an electronic portal imaging device (EPID).^(^ ^12^ ^–^ ^18^ ^)^ Fluence data acquired by the EPID during treatment delivery can be used to reconstruct the dose delivered to the patient on either reference or daily volumetric images. A similar paradigm has been described using ion chambers mounted upstream of the patient.^(^ ^19^ ^,^ ^20^ ^)^ While such techniques have been proven effective, their implementation can be complex, expensive, and laborious for routine use. An alternate strategy involves collecting and analyzing delivery log files directly from the linear accelerator control system.^(^ ^21^ ^–^ ^23^ ^)^ The delivery logs can be used to reconstruct the dose delivered to the patient using actual delivery parameters. However, this process requires diligent, manual collection of data at frequent intervals from the control system, followed by processing and analysis of the data using independent software. Because of these challenges, daily delivery verification of VMAT treatments is left to the linear accelerator vendor at most institutions. However, a third potential approach, which involves using data contained within the record and verify (RV) system, may offer a robust, low‐cost, and automated solution to daily delivery verification of VMAT.

Commercial RV systems, also known as Oncology Information Systems, are comprehensive information management systems that aggregate data into an electronic medical chart. They have gained widespread acceptance at institutions providing radiation therapy. As they pertain to treatment delivery, current RV systems store the planned treatment parameters (as determined by the treatment planning system), transfer them to the linear accelerator treatment control system for delivery, and then record the delivered treatment parameters (as reported by the treatment control system) for each patient and delivery. In theory, this data could be used to verify machine performance and delivery. However, there are no tools in current versions of the RV system to monitor such behavior. Moreover, access to detailed delivery data is limited to visual inspection and manual recording of delivery parameter values.

In this work, we report on an automated software system for daily delivery verification of VMAT treatments. The system was designed to collect all relevant treatment values from the RV system database, analyze the delivery data relative to planned data, and report the results to responsible technical personnel. The utility of the system was investigated through analysis of patient data collected at our clinic.

## II. MATERIALS AND METHODS

### A. VMAT treatment planning and delivery

VMAT was commissioned and released for clinical use at our clinic in February 2010. All VMAT treatment plans are constructed using version 9.0 of the Philips Pinnacle treatment planning system (Fitchburg, WI, USA). VMAT plans are constructed using the SmartArc module,^(5)^ utilize 6 MV beams and a static collimator angle of 45°. All VMAT plans consisted of one or two arcs, beginning at 175° and rotating counter‐clockwise to 185°, with a return arc in the clockwise direction if needed. For SmartArc optimization, the final gantry spacing was set to $\Delta =4^{\circ }$, with the maximum delivery time set to 90 sec per arc and maximum leaf motion constrained to 4 mm per degree of gantry rotation. Following completion of treatment planning, all plans are transferred to a commercial RV system (Mosaiq 1.60, Elekta, Mountain View, CA) to facilitate delivery.

All VMAT clinical plans were delivered using an Elekta Infinity radiotherapy accelerator (Crawley, UK) utilizing the Desktop Pro R7.0x control system. The Elekta Infinity system features an 80‐leaf multileaf collimator (MLC) with 1 cm leaf widths and delivers VMAT plans using discretely variable dose rates (i.e., 500, 250, 125, 63, 37 MU/min) and continuously variable gantry speed.^(24)^ The Desktop Pro 7.0x control system converts a VMAT plan into a delivery sequence by converting control points (which include only gantry angle, MLC shape, and cumulative monitor units) provided by the RV system into a series of deliverable steps (control point pairs) using machine parameters (dose rates, MLC leaf speeds, and gantry speeds) available to the control system at that time. The dose rate is automatically selected by the control system on the basis of the requested movements and the maximum possible speeds in order to deliver each step in the minimum time. The nominal speeds are then calculated on the basis of this dose rate. The actual speeds are servo‐controlled to ensure that all the parameters are within positional tolerance of their planned positions at all times.

During delivery, there were two separate checks to verify the treatment is delivered as planned. First, the control system checks all parameters are within tolerance every 40 ms. The second delivery check operates at control point boundaries and is run outside of the control system. The relevant delivery parameters (e.g., gantry position, leaf positions, jaw positions, and cumulative monitor units) are captured by the control system at each control point boundary and checked against the planned positions. The captured delivery parameters are subsequently reported via the iCom‐Vx network interface to the Mosaiq RV system, which stores the data within the patient's treatment history. The reported data stored within the RV system serves as the basis for the delivery verification system described in the following section.

Prior to clinical delivery of VMAT treatments, all treatment plans were transferred to a cylindrical water phantom (Cheese Phantom, TomoTherapy, Inc., Madison, WI) for the purpose of patient‐specific quality assurance measurements using radiographic film (EDR‐2, Eastman Kodak Co., Rochester, NY) and a calibrated cylindrical ion chamber (A1SL, Standard Imaging, Madison, WI).

### B. Software system configuration

The performance verification software system consists of three main components: (1) a custom database query module to collect and report daily delivery data, (2) an analysis module that reads the daily delivery report generated from the database query, analyzes the data, and compiles treatment error reports, and (3) a graphical reporting module that alerts the user (via email) to possible errors and enables the user to investigate instances of unusual treatment behavior. The database query module is configured as an automated daily report scheduled through a Crystal Reports server (though it should be noted that the system can be configured using any SQL‐compliant code and scheduler). The module is launched following the close of operating hours and queries the Mosiaq SQL database for patients treated using the “VMAT” field tag during that day. For each patient flagged as being treated with VMAT, the module returns the MLC positions, gantry position, and jaw positions reported to the RV system by the linear accelerator control system during VMAT delivery. The module also returns planned parameters from the plan file. All delivery parameters (planned and reported for that day) are reported in the form of a plain text file written to a shared network drive. The daily delivery report is then accessible to anyone with sufficient privileges and access to the institutional network.

The analysis module was written in MATLAB 9 (The MathWorks, Inc., Natick, MA), and utilizes data provided by the database query module as input. The analysis module is also an automated daily task scheduled using the Windows XP (Microsoft Corp., Redmond, WA) desktop scheduler. The analysis module is launched each morning prior to operating hours and reads the daily delivery report generated by the database query module. The analysis module contains a script that reads the daily delivery report and generates a list of patients contained within the report. For each patient, the MLC, gantry and jaw positions reported by the control system during VMAT delivery are compared to the planned positions by linearly interpolating (on the basis of cumulative monitor units) between control point pairs from the planned positions. MLC positions corresponding to leaves blocked by the jaws were excluded from analysis. The script then generates a delivery error log for each patient, tagged with the date of the delivered fraction. The result of the script is a record of MLC, gantry, and jaw position errors for every VMAT fraction delivered that can be sorted by patient and date, and can be subsequently analyzed using any scripting or programming language capable of reading plain text files. Alternatively, the delivery error logs could be reformatted into a separate database structure using commercially available software. The current data analysis module also contains a script that calculates user‐defined metrics, such as percentage of reported MLC positions within 3 mm of their planned positions, and can be configured to notify authorized technical staff via simple mail transfer protocol (i.e., email) of treatment delivery abnormalities or instances where predefined tolerance values are exceeded.

The graphical reporting module was also written in MATLAB 9 and utilizes daily delivery error logs generated by the analysis module as input. The graphical reporting module is launched by the user, and allows access to the delivery error logs for every patient and fraction. The user can display MLC leaf position errors, gantry position errors, and jaw position errors for a single treatment day, for a single patient, or for all treatments delivered. The user can further investigate single‐fraction error log data in a separate window, which displays histograms of the MLC, gantry, and jaw position errors.

### C. Clinical data

**Figure 1:**
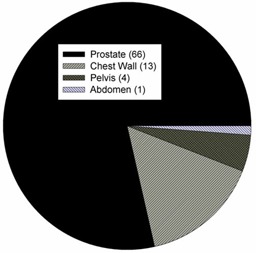
Distribution of anatomical sites treated at our clinic using VMAT. The number in parenthesis indicates the number of patients treated within the site category.

The delivery verification system was launched in July 2010 and has been generating automated daily reports since that time. Before launching the system, the scripts were also backdated to collect delivery data beginning with the first patient VMAT fraction delivered at our center in February 2010. Delivery data has been collected for 84 patients and approximately 2500 fractions. Analysis of every treatment fraction has resulted in the evaluation of more than $2.3\times 10^{7}$ MLC leaf positions, $1.1\times 10^{6}$ jaw positions, and $2.9\times 10^{5}$ gantry positions. The distribution of anatomical sites treated clinically with VMAT at our center is shown in Fig. 1, with prostate being the most common site treated with VMAT at this time.

## III. RESULTS

**Figure 2:**
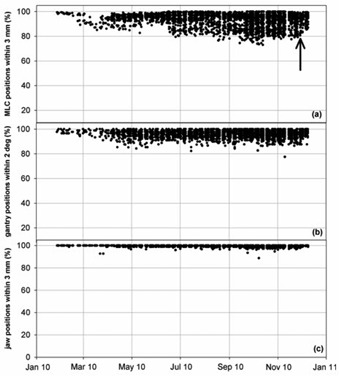
Sample results from the graphical reporting module, displaying historical results for: (a) the percentage of MLC leaf positions within 3 mm, (b) the percentage of gantry positions within 2°, (c) the percentage of jaw positions within 3 mm of their planned positions. Each data point corresponds to a single patient fraction. The arrow indicates the date of adjustments to VMAT‐related calibrations.

Sample results from the graphical reporting module are shown in Fig. 2, which displays historical results for (a) the percentage of MLC leaf positions within 3 mm, (b) the percentage of gantry positions within 2°, and (c) the percentage of jaw positions within 3 mm of their planned positions. Each data point corresponds to a single patient fraction. The average percentage of MLC leaf positions within 3 mm, gantry positions within 2°, and jaw positions within 3 mm of planned positions was 92.9%±5.5%,95.9%±2.9%, and 99.7%±0.6%, respectively (additional results at different thresholds are provided in Table 1). The level of agreement between planned and delivered MLC positions appears to have varied with time and was primarily attributed to increasing plan complexity of treated patients, whereby plans requiring larger MLC leaf movements between control points showed lower levels of agreement with their planned positions (described later in this section). A less prominent but nonetheless notable cause for the time‐varying nature of the MLC position agreement was attributed to changes in calibration settings of the control system. For example, adjustments were made to gantry movement servos, MLC leaf speed calibrations, and dose rate calibration over the course of several evenings in early December 2010. Despite a constant plan complexity profile during the daily clinical schedule, the level agreement for reported MLC positions improved following adjustment of calibration settings (indicated in Fig. 2(a).

**Table 1:** The percentages of MLC, jaw, and gantry positions within various tolerance values for all patients, patients receiving irradiation of the prostatic volume only, and patients receiving irradiation of the chest wall.

	*Tolerance (millimeters or degrees)*			
*Parameter*	*1*	*2*	*3*	*5*
		*All Patients*		
MLC	75.2	87.3	92.9	98.2
Jaw	92.8	99.4	99.7	100
Gantry	83.0	95.9	99.2	100
		*Prostate Patients*		
MLC	84.5	93.4	96.8	99.3
Jaw	95.4	99.7	99.8	100
Gantry	82.3	95.7	99.4	100
		*Chest Wall Patients*		
MLC	54.4	73.2	84.6	95.7
Jaw	84.4	98.1	99.5	100
Gantry	86.6	97.1	99.7	100

**Figure 3:**
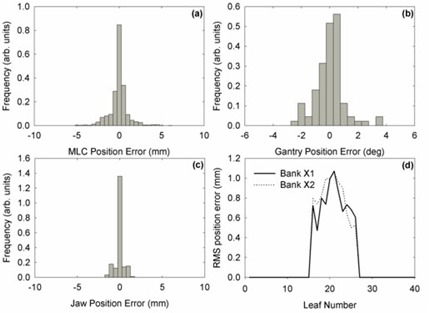
A sample single‐fraction analysis window for a prostate VMAT treatment. Histograms of (a) MLC leaf errors, (b) gantry position errors, (c) jaw position errors, and (d) RMS position errors for each leaf are displayed.

Figure 3 shows a sample single‐fraction analysis window for a prostate VMAT treatment. For the fraction shown, average percentage of MLC leaf positions within 3 mm, gantry positions within 2°, and jaw positions within 3 mm of planned positions was 97.9%, 94.3%, and 100%, respectively.

**Figure 4:**
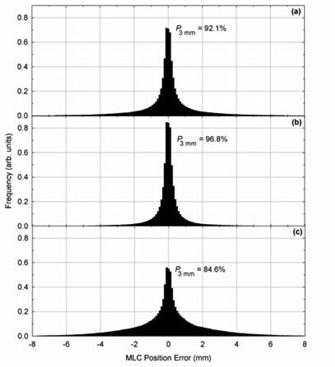
Histograms of MLC position errors for: (a) all patients, (b) patients receiving irradiation of the prostatic volume only, and (c) patients receiving postmastectomy chest wall irradiation. The quantity $P_{3\text{mm}}$ represents the percentage of MLC leaf positions within 3 mm of their planned position.

**Figure 5:**
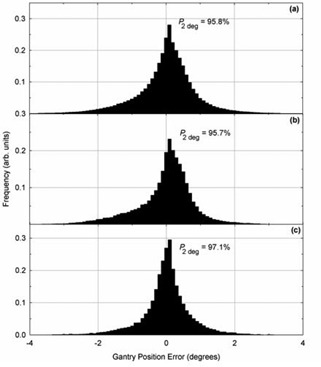
Histograms of gantry position errors for: (a) all patients, (b) patients receiving irradiation of the prostatic volume only, and (c) patients receiving postmastectomy chest wall irradiation. The quantity $P_{2\text{deg}}$ represents the percentage of gantry positions within 2° of their planned position.

As noted above, the level of agreement between planned and delivered positions depended on plan complexity, particularly for reported MLC leaf positions. Targets requiring greater MLC leaf movements between control points showed lower levels of agreement. Furthermore, because MLC leaf movement was observed to depend on target size, the level of agreement between planned and delivered positions could be generalized by site. For example, the average percentage of reported MLC leaf positions within 3 mm of their planned positions was 96.8%±2.2% for patients receiving irradiation of the prostatic volume only, but was 84.6%±2.8% for patients receiving postmastectomy chest wall irradiation. Figures 4 and 5 show position error histograms for MLC positions and gantry positions, respectively. Multifractional analysis of individual patients showed an average standard deviation in the percentage of MLC locations within 3 mm of their planned position of 5.1%(2−σ) for postmastectomy chest wall patients and 3.4%(2−σ) for prostate patients.

**Figure 6:**
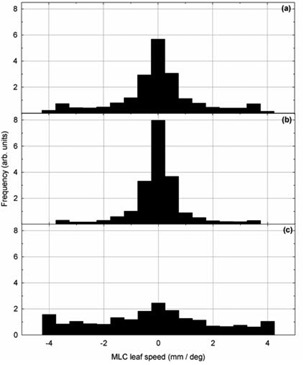
Histograms of planned MLC leaf speeds (expressed in mm of leaf travel per degree of gantry rotation) for: (a) all patients, (b) patients receiving irradiation of the prostatic volume only, and (c) patients receiving postmastectomy chest wall irradiation.

**Figure 7:**
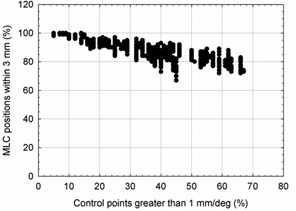
The relationship between leaf movement (shown as the percentage of fractional leaf movements requiring more than 1 mm of travel per degree of gantry rotation) and reported MLC position error (shown as the percentage of reported MLC positions within 3 mm of their planned position). MLC position error was observed to increase in plans with larger MLC leaf movements. Each data point corresponds to a single patient fraction.

Histograms of the planned MLC leaf speeds (expressed in mm of leaf travel per degree of gantry rotation) are shown in Fig. 6 for (a) all patients, (b) patients receiving irradiation of the prostatic volume only, and (c) patients receiving postmastectomy chest wall irradiation. As indicated by the data, postmastectomy chest wall plans required greater MLC leaf movement and were observed to show lower levels of agreement between planned and reported MLC positions. Agreement between planned and reported gantry and jaw positions did not appear influenced by treatment site. Figure 7 shows the relationship between leaf movement and reported MLC position error.

## IV. DISCUSSION

A delivery verification system for VMAT treatments has been developed and implemented using data stored within a commercial RV system. The system automatically generates a delivery error report outlining MLC leaf, gantry, and jaw positions' errors for VMAT treatment each day. The system can display reports initiated by a user anywhere within institutional network, or can be configured to alert authorized users when predefined tolerance values are exceeded. At present, more than 2500 VMAT fractions have been analyzed with the delivery verification system at our institution.

The significance of this work is derived from the need to introduce additional systems that ensure safe and effective patient treatments as radiation therapy treatments become increasingly complex. The delivery verification system described in this work may offer the potential to ensure VMAT treatments are delivered within acceptable tolerances of the planned parameters. Furthermore, the system allows the user to track the performance of the delivery system over time, and may indicate when adjustments to calibration settings are needed. The advantages of this system over other delivery verification systems are: (1) it can be completely automated and accessed remotely; (2) it requires little or no additional effort to utilize once the system has been initiated; and (3) it utilizes commercially‐available products, reducing costs typically associated with additional quality assurance measures.

**Figure 8:**
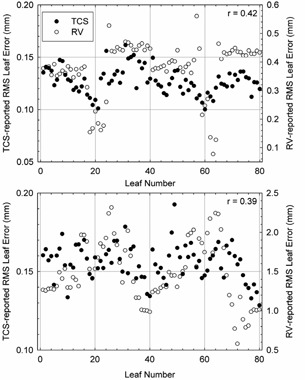
Average MLC leaf errors as reported by the RV‐based delivery verification system (open circles, right axis) and the treatment control system (filled circles, left axis) for a prostate (top) and a chest wall (bottom) fraction. The Pearson product‐moment correlation coefficient (*r*) for each paired dataset is also shown.

Although the delivery verification system of this work holds promise to improve patient safety, there are a number of potential concerns and limitations. Most notably, the level of agreement between planned and reported MLC leaf positions appears larger than tolerances values stated by the manufacturer. The manufacturer specifies a dynamic leaf position tolerance of 1 mm for the MLCi2 head currently in use at our clinic, yet the results of the delivery verification system described in this work show errors in MLC leaf position by as much at 6–7mm. One potential explanation for this discrepancy may be differences in sampling frequency between parameters. During delivery, the MU value is read from the hardware and placed in the memory every 20 ms, whereas the MLC positions are determined from optical reflectors and placed in the memory every 40 ms. There may also be a small additional delay in the treatment control system detection of a control point boundary, which triggers transmission of delivery parameters stored in the memory back to the RV system. The result of this latency may thus overestimate true differences between planned and delivered parameters, particularly in instances of large leaf movements. To examine this effect, MLC position errors reported by the delivery verification system were compared with MLC position errors extracted manually from the treatment control system for one prostate fraction and one chest wall fraction. In general, a systematic difference in the reported MLC position error between the two systems was noted, and was observed to increase in the plan requiring greater leaf movements (see Fig. 8). Averaged over all leaves, the reported MLC position error was 0.38±0.03mm and 0.13±0.003mm for the delivery verification and treatment control systems, respectively, for the prostate patient; and 1.48±0.16mm and 0.16±0.01mm for the for the delivery verification and treatment control systems, respectively, for the chest wall patient. The Pearson product‐moment correlation coefficient (*r*) was used to assess the degree of linear dependence between the two datasets, and yielded a value of approximately 0.4 for both fractions. This suggests a moderate‐to‐strong positive correlation between the results of each system that, due to the number of samples in each dataset (N=80), is unlikely (less than 0.05%) to have resulted from random probability. Based on these observations, we may theorize that the systematic differences between the results of each system can be characterized in future work and appropriately accounted for. Future work notwithstanding, the results nevertheless suggest that the data reported by the delivery verification system may be sufficiently accurate for monitoring and verification of VMAT treatment delivery.

## V. CONCLUSIONS

A remote, automated delivery verification system for VMAT treatments has been developed and implemented using data stored within a commercial RV system. The system generates daily delivery error reports outlining MLC leaf, gantry, and jaw positions' errors for VMAT treatments, and can be configured to alert authorized users when pre‐defined tolerance values are exceeded. The delivery verification system may provide an effective and low‐cost quality assurance tool that improves the safety and effectiveness of every VMAT treatment delivered. Further investigation is needed to confirm the utility of the system.

## ACKNOWLEDGMENTS

The author would like to thank Kevin Brown for providing technical information regarding the Elekta Desktop Pro 7.0x control system, Leon Tomczyk for providing technical information regarding the Elekta Mosaiq recording system, Phillip Atkinson for assisting in developing the SQL database query module, and Mich Price, Ph.D., and Brent Parker, Ph.D., for enlightening and sometimes humorous technical discussions.
